# Migratory Pulmonary Infiltrates Mimicking Malignancy, Tuberculosis, and Sarcoidosis in an Apparently Immunocompetent Adult: A Case of Tissue-Proven Invasive Fungal Disease Due to a Non-*marneffei* *Penicillium* Species

**DOI:** 10.3390/jof12090671

**Published:** 2026-09-07

**Authors:** Houda Gharsalli, Safia Abdullah Mohammed, Abdalla M. Alasiri, Mazen Ahmad Hadi Jali, Nawal Alwadai, Achour Boussafsaf, Nada Abdallah Basheer, Mohannad Saleh AlDossari, Adel Mousa Khedrah, Mohammed Alshahrani, Yahya Shabi

**Affiliations:** 1 Pulmonology Department, Aseer Central Hospital, Abha 62523, Saudi Arabia; houdagharsalli@yahoo.com (H.G.); abokhogmah@hotmail.com (A.M.A.); nana-1866@hotmail.com (N.A.); malshahrani24@moh.gov.sa (M.A.); 2 Infectious Diseases Department, Aseer Central Hospital, Abha 62523, Saudi Arabia; safiaam@moh.gov.sa; 3 Internal Medicine Residency Program, Aseer Central Hospital, Abha 62523, Saudi Arabia; mazen_7750@hotmail.com; 4 Thoracic Surgery Department, Aseer Central Hospital, Abha 62523, Saudi Arabia; achour.bsf30@gmail.com; 5 Microbiology Department, Aseer Central Hospital, Abha 62523, Saudi Arabia; basheer.nada@gmail.com; 6 Histopathology Department, Aseer Central Hospital, Abha 62523, Saudi Arabia; mohnd13548@gmail.com; 7 Radiology Department, Aseer Central Hospital, Abha 62523, Saudi Arabia; akhedrah@moh.gov.sa; 8 Department of Microbiology and Clinical Parasitology, College of Medicine, King Khalid University, Abha 61421, Saudi Arabia

**Keywords:** *Penicillium*, non-*marneffei Penicillium*, invasive fungal disease, migratory pulmonary infiltrates, necrotizing granuloma, apparently immunocompetent host, diagnostic uncertainty, bronchoalveolar lavage, voriconazole

## Abstract

Non-*marneffei Penicillium* species are ubiquitous molds usually dismissed as contaminants in respiratory cultures; invasive disease is rare and may mimic malignancy, tuberculosis, or sarcoidosis. A 39-year-old apparently immunocompetent man with asthma, a smoking history, and tuberculosis exposure presented with chronic cough, fever, night sweats, and weight loss. Chest computed tomography (CT) showed right lower-lobe subpleural consolidation, bilateral nodules, and mediastinal lymphadenopathy. Sputum and bronchoalveolar lavage (BAL) studies for tuberculosis were repeatedly negative; CT-guided lung biopsy showed a non-necrotizing granuloma, suggesting sarcoidosis. Five months later, the original lesions had regressed, and new contralateral pleural-based nodules appeared, establishing a migratory pattern. Repeat BAL grew a *Penicillium* species, a second recovery after an earlier isolate was dismissed as contamination. Surgical mediastinal lymph-node biopsy showed necrotizing granulomatous inflammation containing PAS- and GMS-positive septate hyphae, establishing proven invasive fungal disease, and fungal culture of the same tissue grew a *Penicillium* species; HIV and hepatitis serologies were negative. Liposomal amphotericin B followed by oral voriconazole produced near-complete radiological resolution. Attribution to *Penicillium* rests on recovery of the mold from the surgical tissue itself; identification nevertheless remained at genus level, because molecular identification, susceptibility testing, and fungal biomarkers were unavailable, and invasion of lung parenchyma was never demonstrated histologically. Migratory infiltrates should prompt reconsideration of fixed diagnoses; repeated mold recovery with tissue-invasive hyphae and treatment response warrants reassessment rather than dismissal as contamination.

## 1. Introduction

The genus *Penicillium* comprises ubiquitous filamentous molds found in soil, air, decaying vegetation, indoor environments, and food products [1]. Because airborne conidia are common, *Penicillium* species are frequently recovered as environmental contaminants in clinical cultures, including respiratory specimens [2,3]. With the exception of *Talaromyces marneffei*, formerly classified as *Penicillium marneffei*, invasive disease due to non-*marneffei Penicillium* species is rare and is reported mainly in immunocompromised hosts, including patients with hematologic malignancy, transplantation, advanced HIV infection, chronic granulomatous disease, or other major immune defects [2,3,4,5].

Pulmonary infection caused by non-*marneffei Penicillium* species in apparently immunocompetent adults is distinctly uncommon [2,5]. Diagnosis is challenging because growth in a respiratory culture may reflect contamination of the specimen or colonization of the airway rather than invasive disease [2,3]. Stronger evidence of pathogenicity includes repeated microbiological recovery, exclusion of alternative diagnoses, histopathological demonstration of tissue-invasive fungal elements, and clinical-radiological response to antifungal therapy [3,6].

We report an apparently immunocompetent adult with tissue-proven invasive fungal disease of the thorax, involving lung and mediastinal lymph nodes, in whom repeated respiratory recovery of a *Penicillium* species, necrotizing granulomatous inflammation containing fungal hyphae, exclusion of tuberculosis, malignancy and sarcoidosis, recovery of the same mold in culture from the surgically obtained tissue, and response to antifungal therapy together identify a *Penicillium* species as the pathogen. Because the organism was identified only to genus level, we present the genus attribution as established while its species identity remains undetermined.

## 2. Case Presentation

### 2.1. Initial Presentation (Index Admission)

A 39-year-old man presented to the emergency department with a productive cough that had begun approximately one year earlier and had worsened markedly over the preceding three months, with acute deterioration in the final ten days. He produced moderate amounts of white sputum without hemoptysis and reported drenching night sweats, an estimated 5–10 kg of weight loss over three months, intermittent fever (documented to 38 °C, responsive to antipyretics), and episodes of post-tussive vomiting. He had bronchial asthma, diagnosed more than a decade earlier and controlled with inhaled salbutamol and budesonide, with infrequent exacerbations and no prior intensive-care admission. He was a current smoker, used cannabis (hashish), and had been incarcerated for 1.5 years, during which he reported three known contacts with patients who had active pulmonary tuberculosis.

On examination he was well-appearing, alert and fully oriented. He was hemodynamically stable with oxygen saturation 94% on room air, blood pressure 116/81 mmHg and heart rate 98 beats/min. He developed self-limiting epistaxis during the assessment. Chest auscultation revealed bilateral crepitations with equal air entry. The abdomen was soft and non-tender with no organomegaly, and the remainder of the examination was unremarkable.

### 2.2. Initial Investigations

Laboratory results are summarized in Table 1. Of note, renal function was preserved; there was a mild transaminitis and a reactive thrombocytosis, and the C-reactive protein was normal. Screening serologies for HIV, hepatitis B and hepatitis C were negative. An interferon-gamma release assay (IGRA) for *Mycobacterium tuberculosis* was reactive, consistent with latent TB infection in the context of documented exposure.

**Figure 1:**
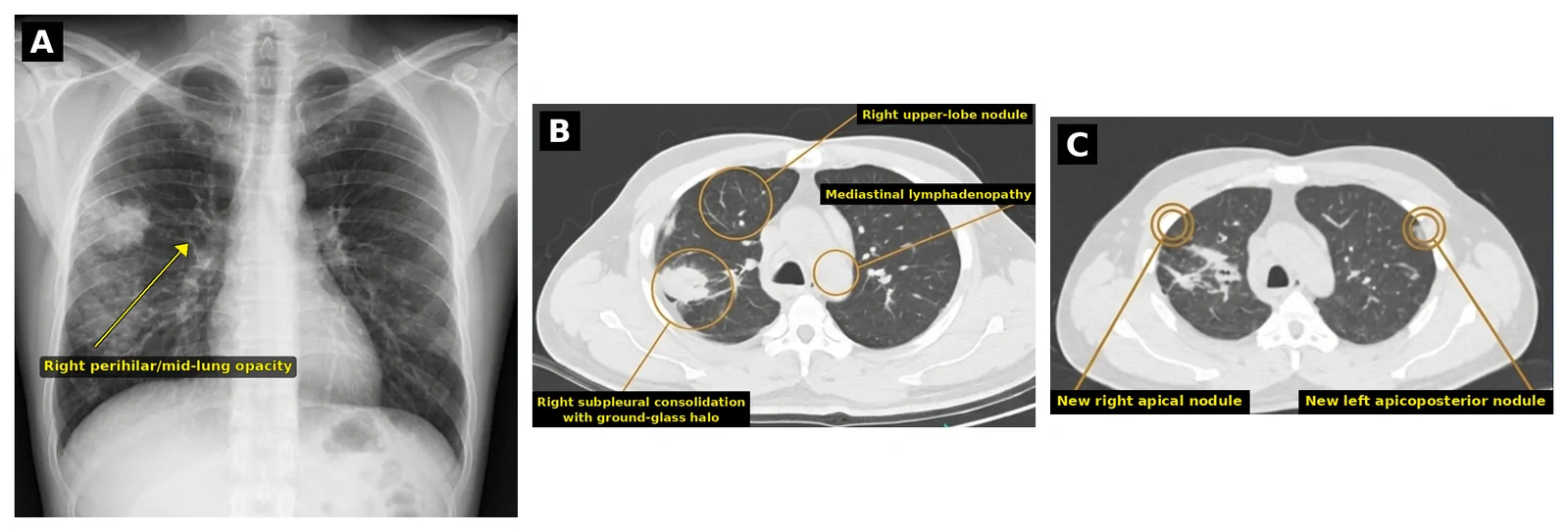
Serial thoracic imaging. (**A**) Posteroanterior chest radiograph at index presentation: a rounded right perihilar/mid-lung opacity (arrow). (**B**) Axial contrast-enhanced chest computed tomography (CT) at index presentation, showing a large right-sided subpleural consolidation with a peripheral ground-glass halo, a right upper-lobe nodule, and subcarinal lymphadenopathy; the circles mark these three findings and are labeled individually on the panel. (**C**) Axial contrast-enhanced chest CT at readmission, approximately five months later, showing interval regression of the original right-sided lesion with new bilateral pleural-based upper-lobe nodules (labeled arrows: a new right apical nodule and a new left apicoposterior nodule), consistent with a migratory pattern. Panels (**B**,**C**) are axial 1.25 mm sections from contrast-enhanced chest CT acquired on the same scanner using the same institutional protocol, displayed on a lung window (window width 1500 HU, window level −600 HU).

Initial chest radiography showed a suspicious right-sided pulmonary opacity, prompting contrast-enhanced chest CT, which demonstrated a 7.5 × 4.5 cm right lower-lobe subpleural consolidation with a peripheral ground-glass halo, multiple bilateral nodules, and subcarinal lymphadenopathy measuring 2.7 cm (Figure 1A,B). Given the clinical history, the reporting radiologist considered the findings highly concerning for primary lung malignancy (adenocarcinoma) with metastases, with pulmonary tuberculosis as the principal alternative.

### 2.3. Index Admission Course and the First Diagnostic Detour

An infectious, and specifically mycobacterial, etiology was pursued aggressively, and the specimens and assays used are enumerated here in full. Three separate expectorated sputum specimens were negative for acid-fast bacilli (AFB) on smear microscopy and negative by polymerase chain reaction (PCR) using the Xpert MTB/RIF Ultra assay (Cepheid, Sunnyvale, CA, USA), and sputum fungal culture showed no growth after four weeks. Flexible bronchoscopy was macroscopically unremarkable; bronchoalveolar lavage (BAL) fluid was negative for AFB on smear microscopy and negative for *Mycobacterium tuberculosis* complex by Xpert MTB/RIF Ultra PCR, and bacterial culture showed normal flora. A CT-guided percutaneous biopsy of the lung parenchyma demonstrated a non-necrotizing epithelioid granuloma, with negative special stains and cultures. Mycobacterial culture was requested on all three expectorated sputum specimens, the bronchoalveolar lavage fluid, the CT-guided core-biopsy tissue and the surgical mediastinal lymph-node tissue, and cultures were incubated for 6 weeks in liquid (MGIT) medium and 8 weeks on Löwenstein–Jensen solid medium in accordance with standard recommendations for mycobacterial culture [7]; all remained negative at the end of incubation.

Given the bilateral nodular disease, repeatedly negative mycobacterial work-up, and non-necrotizing granuloma on core biopsy, the overall picture was interpreted as most consistent with sarcoidosis. The patient was discharged with a presumptive diagnosis of sarcoidosis and a plan for outpatient pulmonology follow-up and further work-up. With respect to immunosuppressive therapy for the presumptive sarcoidosis, no systemic corticosteroid or other immunosuppressive agent was prescribed at any point during or after this admission. Treatment of latent tuberculosis infection was considered but deferred pending completion of the outpatient sarcoidosis work-up. Critically, he was subsequently lost to follow-up, so neither the planned outpatient sarcoidosis work-up nor any latent tuberculosis regimen could be supervised or documented as completed [8]. A mold morphologically identified as a *Penicillium* species was also recovered during this index admission from a lower respiratory tract specimen recorded as an endotracheal aspirate obtained at the flexible bronchoscopy performed during the same admission; the patient was not intubated at any point, and the specimen is correctly designated a bronchial aspirate rather than an endotracheal aspirate. The culture became positive after 4 days of incubation. Lactophenol cotton blue microscopy was performed on this isolate and showed hyaline septate hyphae bearing brush-like (penicillate) conidiophores with metulae and flask-shaped phialides producing chains of conidia, consistent with a *Penicillium* species. Because *Penicillium* species are ubiquitous airborne contaminants, because the growth came from a single non-sterile respiratory specimen, and because the working diagnosis at that time was malignancy or tuberculosis, the result was interpreted as contamination and was not acted upon. On retrospective review of the laboratory records, the index-admission isolate and the later bronchoalveolar lavage isolate were comparable in both colony morphology and microscopic appearance, although in the absence of sequencing they cannot be shown to belong to the same species. The two growths came from separate specimens obtained approximately five months apart and therefore represent two independent recoveries rather than a single contaminated specimen.

### 2.4. Readmission and the Diagnostic Pivot

The migratory behavior of the lesions became the diagnostic turning point. Regression of the original right-sided lesions with emergence of new contralateral pleural-based nodules was not compatible with an untreated fixed solid malignancy and argued against anchoring on sarcoidosis alone (Table 2; Figure 1C). The differential shifted toward migratory inflammatory or infectious lung disease, including organizing pneumonia, eosinophilic pneumonia, vasculitis, tuberculosis, and invasive fungal infection.

### 2.5. Repeat Bronchoscopy and Bronchoalveolar Lavage

**Figure 2:**
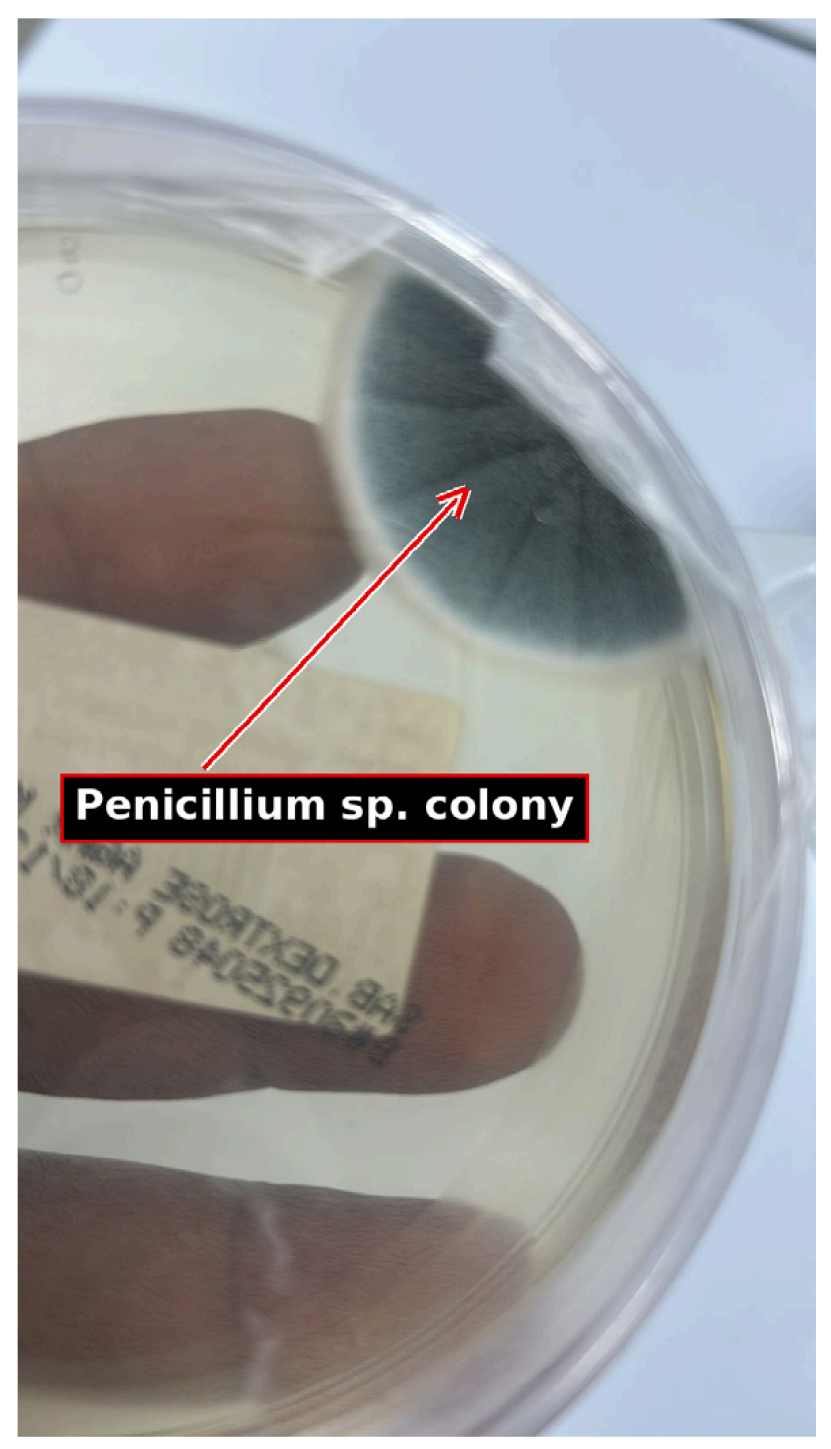
Fungal culture of bronchoalveolar lavage fluid on Sabouraud dextrose agar, photographed after 5 days of incubation at 30 °C: a gray-green velvety mold colony with a pale peripheral margin (arrow), consistent with a *Penicillium* species. Colony morphology alone does not permit identification below genus level.

Repeat bronchoscopy showed multifocal bronchial narrowing but no endobronchial mass. BAL fluid was reddish and turbid, with 2,860 white cells/µL and a differential of 70% macrophages, 20% lymphocytes, and 10% neutrophils. BAL smear and PCR for tuberculosis were again negative. Fungal culture of the bronchoalveolar lavage fluid grew a mold morphologically consistent with a *Penicillium* species (Figure 2), whereas the concurrent sputum fungal culture showed no growth.

### 2.6. Mycological Identification

All mycological work was performed in the clinical microbiology laboratory of Aseer Central Hospital according to its standard operating procedures for filamentous fungi. Bronchoalveolar lavage fluid was inoculated onto Sabouraud dextrose agar with chloramphenicol as the sole medium and incubated at 30 °C and held for four weeks before a negative report was issued; consistent with this practice, the index-admission sputum fungal culture had been reported as showing no growth only after four weeks. Growth of the bronchoalveolar lavage isolate first became visible on day 3. The mature colony was gray-green and velvety with a pale periphery on the obverse (Figure 2), and the reverse was pale to cream, with no diffusible pigment. Microscopic examination was performed using a lactophenol cotton blue wet mount and demonstrated hyaline septate hyphae bearing brush-like (penicillate) conidiophores with metulae and flask-shaped phialides producing chains of smooth-walled, globose to subglobose conidia, a morphology that defines the genus *Penicillium* but does not identify a species. Matrix-assisted laser desorption/ionization time-of-flight mass spectrometry (MALDI-TOF MS) was not available in our laboratory at the time. Performance of MALDI-TOF MS for filamentous fungi is in any case constrained by reference-database coverage: with commercial libraries alone only a minority of mold isolates reach a reliable species-level identification, and supplementation with in-house spectra is generally required [9]. Sequencing of the internal transcribed spacer (ITS) region and of the secondary markers used for the Eurotiales, beta-tubulin (BenA) and calmodulin (CaM), was not performed because fungal sequencing is not offered in-house, and referral of the isolate to a reference mycology laboratory could not be funded at the time. This is a substantive limitation, because species boundaries within *Penicillium* and the closely related genus *Talaromyces* are defined phylogenetically and are not reliably resolved by phenotype: multilocus sequence analysis of ITS, BenA, CaM and RPB2 is the accepted standard for identifying *Penicillium*-like fungi, including clinical isolates [1,10,11]. Antifungal susceptibility testing was likewise unavailable. The isolate was discarded after routine reporting and was not stored, so retrospective sequencing is no longer possible. The organism is therefore reported here at genus level only, as a *Penicillium*-like mold most consistent with a *Penicillium* species.

### 2.7. Multidisciplinary Evaluation and Tissue Diagnosis

A multidisciplinary assessment was undertaken. Rheumatology found no evidence of systemic vasculitis; nephrology evaluated a metabolic acidosis and raised the possibility of renal tubular acidosis; cardiology reported an unremarkable electrocardiogram and echocardiogram; and ophthalmology found no uveitis. To overcome the sampling limitation of the earlier core biopsy, thoracic surgery performed a surgical mediastinal lymph-node biopsy.

**Figure 3:**
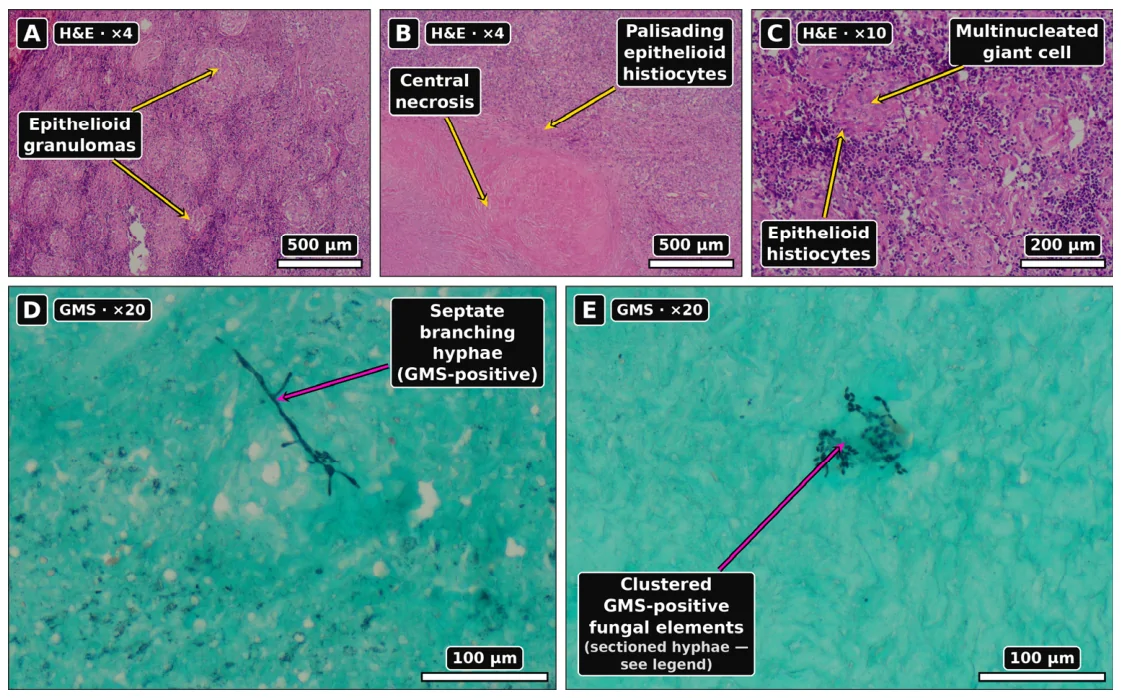
Histopathology of the surgical mediastinal lymph-node biopsy. All five fields are from the same specimen. (**A**) Hematoxylin and eosin (H&E); the two arrows indicate epithelioid granulomas. (**B**) H&E; a necrotizing granuloma, with the upper arrow indicating the palisading rim of epithelioid histiocytes and the lower arrow the central necrosis. (**C**) H&E; the upper arrow indicates a multinucleated giant cell and the lower arrow the surrounding epithelioid histiocytes. (**D**) Grocott methenamine silver (GMS); the arrow indicates septate, branching fungal hyphae. (**E**) GMS; the arrow indicates a cluster of rounded to ovoid GMS-positive fungal structures. These structures are reported descriptively: unless a conidiophore or phialide can be demonstrated bearing them in continuity within the same plane of section, GMS-positive round profiles cannot be distinguished with confidence from transversely or tangentially sectioned hyphae, hyphal swellings, or degenerate fungal elements, and they are therefore not asserted here to be tissue-phase conidia. Original magnification: (**A**) ×4, (**B**) ×4, (**C**) ×10, (**D**) ×20, (**E**) ×20. Scale bars: (**A**) 500 µm, (**B**) 500 µm, (**C**) 200 µm, (**D**) 100 µm, (**E**) 100 µm; in every panel the bar is drawn at exactly 25.0% of the horizontal field width.

In contrast to the non-necrotizing granuloma seen in the first specimen, the surgical biopsy showed a well-formed necrotizing granuloma, with palisading histiocytes and giant cells surrounding central necrosis (Figure 3A–C). Periodic acid–Schiff (PAS) and Grocott methenamine silver (GMS) stains were positive for fungal elements, and staining for acid-fast bacilli was negative. Histopathological examination demonstrated septate, branching fungal hyphae highlighted by PAS and GMS staining within areas of granulomatous inflammation and necrosis (Figure 3D). Clusters of rounded to ovoid GMS-positive fungal structures were also present (Figure 3E). Because no conidiophore or phialide bearing these structures could be demonstrated in continuity within the same plane of section, they are reported descriptively rather than as conidia; on this evidence they cannot be distinguished from transversely or tangentially sectioned hyphae, hyphal swellings, or degenerate fungal elements [12]. The demonstration of septate hyphae within necrotic granulomatous tissue established tissue invasion, and therefore proven invasive fungal disease, which the consensus definitions of the European Organization for Research and Treatment of Cancer and the Mycoses Study Group Education and Research Consortium (EORTC/MSGERC) recognize on histopathological grounds irrespective of host immune status [6]. Tissue morphology could not, however, identify the organism, because hyaline septate hyphae are shared by *Penicillium*, *Talaromyces*, *Aspergillus* and other genera [12]. Fungal culture was, however, set up on the surgical mediastinal lymph-node tissue and grew a *Penicillium* species whose colony and microscopic morphology matched the two respiratory isolates. A portion of the fresh surgical lymph-node tissue was submitted to the mycology bench, minced in sterile saline and inoculated onto Sabouraud dextrose agar with chloramphenicol, and then incubated at 30 °C and held for four weeks, following the same protocol applied to the respiratory specimens; growth became visible after 3 days of incubation, and the isolate was identified by lactophenol cotton blue microscopy using the criteria set out in Section 2.6. Recovery of the organism from surgically obtained tissue that also contained GMS-positive septate hyphae attributes the proven invasive fungal disease to a *Penicillium* species directly, rather than by inference from respiratory isolates alone. The contrast between the two biopsy attempts is summarized in Table 3.

It is useful to separate two levels of diagnostic certainty in this case. First, invasive fungal disease is proven: septate fungal hyphae were demonstrated within necrotizing granulomatous inflammation in surgically obtained tissue, which satisfies the histopathological criterion for proven invasive fungal disease [6]. Second, attribution of that disease to a *Penicillium* species is likewise established and rests on direct as well as converging evidence: a mold morphologically consistent with a *Penicillium* species was recovered from two separate respiratory specimens obtained approximately five months apart and, in culture, from the surgical mediastinal lymph-node tissue itself; repeated sputum, BAL and tissue studies for mycobacteria, including acid-fast staining, PCR and culture, were negative; neither biopsy showed malignancy; the demonstration of an infectious cause of granulomatous inflammation removed the basis for a diagnosis of sarcoidosis, which requires exclusion of alternative causes [13]; and the radiological abnormalities regressed on antifungal therapy. What remains undetermined is the species identity of the organism, not its attribution, and this report maintains that distinction throughout.

Because tissue morphology alone cannot assign the organism to genus level [12], the genus attribution rests on culture of the surgically obtained tissue, together with the converging clinical, microbiological and radiological evidence, rather than on the appearance of the fungal elements in tissue. Taken together, the findings establish proven invasive fungal disease of the thorax due to a non-*marneffei Penicillium* species, with pulmonary and mediastinal nodal involvement, while the species identity remains undetermined [2,3,6].

Two thoracic compartments were involved, and the evidence for each is of a different kind. The parenchymal lung disease was defined radiologically, by the consolidation, the bilateral nodules and their subsequent migration, and microbiologically, by two recoveries of the mold from lower respiratory tract specimens; it was not defined histologically, because the CT-guided core biopsy of lung parenchyma showed only a non-necrotizing granuloma with negative special stains. Tissue proof of fungal invasion came instead from the draining mediastinal lymph node, which also yielded the organism in culture. We therefore describe this case as invasive fungal disease of the thorax with pulmonary and mediastinal nodal involvement, rather than as tissue-proven fungal pneumonia, and we state explicitly that direct histological demonstration of fungal invasion of lung parenchyma was not obtained.

### 2.8. Treatment and Outcome

The patient received induction therapy with intravenous liposomal amphotericin B at a dose of 5 mg/kg daily for 2 weeks. Renal function, serum electrolytes, and liver function tests were monitored throughout treatment. Baseline serum creatinine was 0.82 mg/dL (Table 1); during induction, renal function remained within the reference range and no potassium or magnesium replacement was required. Antifungal susceptibility testing was not available; therefore, treatment was individualized after multidisciplinary discussion and guided by published experience with invasive *Penicillium* infections [2,3,4,5,14,15]. Following clinical stabilization, the patient was transitioned to oral voriconazole 200 mg twice daily after an oral loading dose of 400 mg every 12 h for the first 24 h; oral therapy was continued for a total of 12 weeks, and the planned course was completed.

Hepatic tolerance required particular attention because the patient already had a mild transaminitis at presentation (alanine aminotransferase 112 U/L, aspartate aminotransferase 73 U/L, and gamma-glutamyl transferase 102 U/L; Table 1) and voriconazole is a recognized cause of dose-related hepatotoxicity. Liver chemistry was therefore followed serially during azole therapy, and all three normalized by week 6 without dose reduction or interruption of therapy. Voriconazole therapeutic drug monitoring was not available at our institution; trough-guided dosing is recommended for voriconazole because exposure is highly variable between patients and both subtherapeutic concentrations and concentration-dependent hepatic and neurological toxicity are common [16]. No adverse effects were reported in this patient, including visual disturbance, photosensitivity, hepatotoxicity, and neurological or psychiatric symptoms.

Follow-up chest imaging demonstrated marked interval improvement with near-complete resolution of the migratory nodular and consolidative lesions, and symptoms improved in parallel. After the imaging response was documented there was no further follow-up: having completed the planned course, the patient did not attend his subsequent scheduled appointments and was again lost to follow-up, so no post-treatment surveillance imaging is available.

## 3. Discussion

This case illustrates the following three intertwined diagnostic challenges: the interpretation of migratory pulmonary infiltrates, the limitations of small-volume tissue sampling in granulomatous lung disease, and the perennial difficulty of distinguishing fungal contamination or colonization from true invasive infection, here compounded by an organism—*Penicillium* species—that is reflexively dismissed as an environmental contaminant.

### 3.1. Migratory Infiltrates as a Diagnostic Signpost

The most informative feature of this case was temporal rather than cross-sectional. A lesion that regresses in one region while new lesions appear elsewhere is not typical of untreated fixed solid malignancy and should prompt reassessment of the working diagnosis. The differential diagnosis of migratory pulmonary infiltrates includes organizing pneumonia, eosinophilic pneumonia, allergic bronchopulmonary fungal disease, vasculitis, parasitic infection, tuberculosis, and invasive fungal disease. In this case, direct comparison of serial CT scans dislodged the initial diagnostic anchoring on malignancy and sarcoidosis and redirected the work-up toward infection.

### 3.2. Granuloma Type and Sampling Error

The first biopsy showed a non-necrotizing granuloma, which supported sarcoidosis but did not exclude infection. Current sarcoidosis diagnostic guidance emphasizes that sarcoidosis requires compatible clinical-radiological findings, histological evidence of non-necrotizing granulomatous inflammation, and exclusion of alternative causes of granulomatous disease [13]. In this case, the later surgical biopsy changed the interpretation by demonstrating necrotizing granulomatous inflammation with PAS- and GMS-positive fungal hyphae. This discrepancy highlights the risk of sampling error in granulomatous lung disease, and the consequence is not only diagnostic but therapeutic: a presumptive diagnosis of sarcoidosis carries an implicit indication for systemic corticosteroids, which, in an unrecognized invasive mold infection, would be expected to accelerate rather than control the disease. In the present case no systemic immunosuppression was administered, so the five-month interval between the two admissions represents the natural history of untreated infection and the host can still reasonably be described as apparently immunocompetent. This risk is a further argument for obtaining diagnostic-quality tissue, and for repeating sampling when the clinical course diverges from the working diagnosis, before committing a patient to immunosuppression.

### 3.3. Penicillium as a Pathogen, Not Merely a Contaminant

*Penicillium* species are often dismissed as contaminants because of their environmental ubiquity [1,2,3]. However, invasive infection is increasingly recognized, especially in vulnerable hosts, and pulmonary involvement is among the reported presentations [2,3,4,5,17]. Respiratory isolation alone is insufficient to diagnose invasive disease, and the two questions that follow such an isolate must be kept apart. The first is whether invasive fungal disease is present at all. That question is answered by histopathology as follows: the EORTC/MSGERC consensus definitions treat the demonstration of hyphae with associated tissue damage as proof of invasive fungal disease irrespective of host immune status [6], and in this case that criterion was met in a surgical mediastinal lymph-node specimen. The second question is which organism caused it, and histopathology cannot answer it, because hyaline septate hyphae in tissue are shared by *Penicillium*, *Talaromyces*, *Aspergillus* and other genera [12]. Here, that question was answered by culture rather than by morphology as follows: fungal culture of the surgical mediastinal lymph-node tissue that contained the GMS-positive hyphae grew a *Penicillium* species, and this was supported by two independent respiratory recoveries of a morphologically comparable mold, by the exclusion of tuberculosis, malignancy and sarcoidosis and by radiological response to antifungal therapy. We regard this as establishing a *Penicillium* species as the pathogen, while noting that the organism was never identified directly in tissue by microscopy and that its species identity remains undetermined.

The rounded GMS-positive structures illustrated in Figure 3E are deliberately reported descriptively rather than as conidia. Molds of this group grow in infected tissue as hyphae; conidiogenesis requires an air interface, and sporulation within a tissue section is an uncommon finding [18], described mainly where fungus grows into an air-filled space such as a pulmonary cavity, a bronchial lumen, or a paranasal sinus [19]. A surgically excised mediastinal lymph node offers no such interface, and round profiles in section are far more often transversely or tangentially cut hyphae, hyphal swellings, or degenerate fungal elements. Because the morphological features of fungi in tissue are only rarely specific enough to assign a genus [12], the histopathology in this case is best reported descriptively, and the attribution to *Penicillium* rests on culture of the surgical tissue itself, together with repeated respiratory isolation and the tissue evidence of invasion, rather than on the appearance of the fungal elements themselves.

Serum and BAL fungal biomarkers were not obtained in this patient: galactomannan and beta-D-glucan were not available at our institution at the time of this admission. The omission is worth explaining rather than simply recording, because the two assays address different questions and neither would have settled this case. Galactomannan is an *Aspergillus*-directed antigen assay; it is neither designed nor validated for *Penicillium*, and *Penicillium* species are in fact among the molds documented to cross-react in the assay, so a positive result would have been non-specific and a negative result would not have excluded invasive *Penicillium* disease [20]. Beta-D-glucan, by contrast, is a pan-fungal cell-wall marker with moderate sensitivity and specificity for invasive fungal disease as a class; a positive result would have added supportive evidence that invasive fungal disease was present but would not have identified the genus, and it therefore could not have attributed the disease to *Penicillium* [21]. Neither assay would have substituted for the histopathological demonstration of tissue-invasive hyphae, which remains the criterion on which proven invasive fungal disease rests and which was ultimately what established the diagnosis here [6].

Antifungal therapy remains difficult to standardize because invasive non-*marneffei Penicillium* infections are rare and species-level susceptibility varies. Amphotericin B, itraconazole, voriconazole, echinocandins, and other agents have been used in reported cases, but there is no universally accepted regimen or duration [2,3,5,15]. Published susceptibility data show variable azole activity among *Penicillium*-like fungi, which makes species-level identification and antifungal susceptibility testing important whenever they can be obtained [3,14]. Neither was obtainable here, which is why the regimen used in this patient is reported as what was done rather than as what should be done.

### 3.4. Local Host Factors

Although the patient had no recognized systemic immunodeficiency, host defense is not strictly binary, and several exposures present in this case are worth describing—but describing is all that a single case permits. Chronic tobacco and cannabis (hashish) smoking, long-standing asthma, and prolonged inhaled corticosteroid use have each been discussed in the literature as influences on local airway mucosal defense, and we therefore list them here as possible facilitating factors that were present in this patient, not as identified causes. No causal inference can be drawn from one patient: the exposures were concurrent and cannot be separated from one another, there is no unexposed comparator, and invasive mold disease occurs in people with none of them. We accordingly make no claim that these factors produced the infection, and we would caution against reading the association in that direction.

One observation is nonetheless worth recording as hypothesis-generating rather than explanatory. Cannabis herbal material has been shown to carry viable environmental fungi, including molds, on its leaves and flowers, so that smoking delivers this material directly to the lower airway [22]. That raises an untested possibility—not a demonstrated mechanism—that hashish smoking acted as a route of fungal inoculum delivery in this patient, while its combustion products acted on the same mucosal surface. Distinguishing coincidence from contribution would require systematic exposure data across series of patients with invasive mold disease, which a single report cannot supply; we offer the observation only so that it can be tested in larger cohorts.

### 3.5. Limitations

This report has important limitations. The isolate was identified only to genus level, on the basis of phenotypic characteristics alone; sequencing of the internal transcribed spacer, beta-tubulin and calmodulin loci was not available. Therefore, species-level confirmation and exclusion of closely related taxa, in particular *Talaromyces*, could not be undertaken, and the organism is most accurately described as a *Penicillium*-like mold consistent with a *Penicillium* species [1,10,11]. Because the isolate was not stored after routine reporting, retrospective molecular identification is no longer possible. Antifungal susceptibility testing was also unavailable, which limits certainty regarding the optimal oral step-down agent. Serum and BAL fungal biomarkers, including galactomannan and beta-D-glucan, were not obtained, for the reason given in the Discussion; as noted there, galactomannan is not validated for *Penicillium* and beta-D-glucan does not identify the genus, so neither assay would have replaced histopathological confirmation [20,21]. Comprehensive immunological evaluation was not performed, so although routine testing revealed no systemic immunosuppression, subtle immune defects cannot be excluded; the patient is accordingly described throughout this report as apparently immunocompetent rather than immunocompetent. No systemic corticosteroid or other immunosuppressive therapy was administered at any stage, so the description is not further qualified on that account. A further limitation concerns follow-up: the patient was lost to follow-up on two occasions, once after the index admission and again after completion of antifungal therapy, so neither the durability of the radiological response nor the absence of late relapse could be documented. A further limitation is anatomical: tissue proof of fungal invasion was obtained from a mediastinal lymph node, and invasion of lung parenchyma was never demonstrated directly, so the pulmonary component of the illness remains radiologically and microbiologically, rather than histologically, defined. Taken together, these limitations constrain the species diagnosis rather than the attribution: recovery of the organism from the surgically obtained tissue, together with the tissue evidence of invasion, the exclusion of tuberculosis, malignancy and sarcoidosis, and the response to antifungal therapy, establish a *Penicillium* species as the pathogen, while its species identity remains undetermined.

## 4. Conclusions

Migratory pulmonary infiltrates should prompt reconsideration of a fixed diagnosis such as malignancy or sarcoidosis, and in granulomatous lung disease a single non-necrotizing core biopsy does not exclude infection. In this patient, surgical tissue established proven invasive fungal disease of the thorax; the same tissue yielded a non-*marneffei Penicillium* species in culture; identification nevertheless remained at genus level on phenotypic grounds, and invasion of lung parenchyma was not directly shown. The transferable message is correspondingly narrower than a species diagnosis: where a mold ordinarily regarded as a contaminant is recovered repeatedly from the respiratory tract of a patient who has tissue-invasive fungal disease, a compatible clinical and radiological course, and no better explanation, that recovery deserves careful reassessment rather than automatic dismissal as contamination. Species-level identification and antifungal susceptibility testing should be sought whenever they can be obtained, since they are what would convert such an attribution into a species diagnosis. The antifungal regimen used here was individualized for a single patient after multidisciplinary discussion and is not offered as a recommendation; no treatment inference should be drawn from one case.

## Figures and Tables

**Table 1:** Initial laboratory investigations at index presentation.

Category	Result/Finding
Renal function	Creatinine 0.82 mg/dL (preserved)
Liver function	ALT 112 U/L, AST 73 U/L, GGT 102 U/L (mild transaminitis)
Complete blood count	WBC 9.02 × 10^9^/L; Hb 13.3 g/dL; platelets 498 × 10^9^/L (reactive thrombocytosis)
Inflammatory markers	CRP 3.93 mg/L (normal)
Serology	HIV negative; HBV negative; HCV negative
TB immunology	Interferon-gamma release assay (IGRA): reactive (latent TB infection)

Abbreviations: ALT, alanine aminotransferase; AST, aspartate aminotransferase; CRP, C-reactive protein; GGT, gamma-glutamyl transferase; Hb, hemoglobin; HIV, Human immunodeficiency virus; HBV, hepatitis B virus; HCV, hepatitis C virus; TB, tuberculosis; WBC, white blood cells. Reference ranges per local laboratory.

**Table 2:** Comparison of CT chest findings demonstrating the migratory pattern.

Feature	Index CT (September 2025)	Readmission CT (February–March 2026)
Right lower lobe	7.5 × 4.5 cm subpleural consolidation with peripheral ground-glass halo	Interval regression of previously described right-sided peripheral lesions
Upper lobes	Multiple bilateral nodules; largest right-upper-lobe nodule 3.6 × 3.0 cm	New bilateral pleural-based masses: right apical 16 × 22 × 13 mm; left apicoposterior 8 × 7 × 7 mm (plus two foci)
Lymph nodes	Subcarinal lymphadenopathy 2.7 cm	Interval regression of bilateral hilar and subcarinal nodes
Radiological impression	Highly concerning for primary lung malignancy vs. pulmonary TB	Migratory pattern favoring non-neoplastic inflammatory process; eosinophilic disease vs. organizing pneumonia

Abbreviations: CT, computed tomography; TB, tuberculosis.

**Table 3:** Diagnostic yield of core-needle lung versus surgical mediastinal lymph-node biopsy.

	Attempt 1 (September 2025)	Attempt 2 (March 2026)
Method	CT-guided percutaneous core biopsy (lung parenchyma)	Surgical mediastinal lymph-node biopsy
Histopathology	Non-necrotizing epithelioid granuloma	Well-formed necrotizing granuloma (palisading histiocytes, giant cells around necrosis)
Special stains/culture	Special stains negative; fungal culture no growth	PAS and GMS positive for fungal hyphae; AFB negative; fungal culture of the same tissue grew a *Penicillium* species
Interpretation	Misleading trajectory (suspected sarcoidosis)	Invasive fungal disease proven; attribution to a *Penicillium* species established by culture of the same tissue; species identity undetermined
Mycobacterial culture on tissue	No growth at 8 weeks	No growth at 8 weeks

Abbreviations: AFB, acid-fast bacilli; CT, computed tomography; GMS, Grocott methenamine silver; PAS, periodic acid–Schiff.

## Data Availability

The original contributions presented in this study are included in the article; further inquiries can be directed to the corresponding author.

## Abbreviations

AFB, acid-fast bacilli; ALT, alanine aminotransferase; AST, aspartate aminotransferase; BAL, bronchoalveolar lavage; CRP, C-reactive protein; CT, computed tomography; EORTC, European Organization for Research and Treatment of Cancer; GGT, gamma-glutamyl transferase; GMS, Grocott methenamine silver; H&E, hematoxylin and eosin; HBV, hepatitis B virus; HCV, hepatitis C virus; HIV, human immunodeficiency virus; IGRA, interferon-gamma release assay; ITS, internal transcribed spacer; MALDI-TOF, matrix-assisted laser desorption/ionization time-of-flight; MGIT, mycobacteria growth indicator tube; MSGERC, Mycoses Study Group Education and Research Consortium; PAS, periodic acid–Schiff; PCR, polymerase chain reaction; TB, tuberculosis; TDM, therapeutic drug monitoring.
